# DNA sequence context as a marker of CpG methylation instability in normal and cancer tissues

**DOI:** 10.1038/s41598-020-58331-w

**Published:** 2020-02-03

**Authors:** Giovanni Scala, Antonio Federico, Domenico Palumbo, Sergio Cocozza, Dario Greco

**Affiliations:** 1 Faculty of Medicine and Health Technology, Tampere University, Tampere, Finland; 2 Department of Molecular Medicine and Medical Biotechnology, University of Naples “Federico II”, Naples, Italy; 3 BioMediTech Institute, Tampere University, Tampere, Finland; 4 Institute of Biotechnology, University of Helsinki, Helsinki, Finland; 5 Present Address: Department of Biology, University of Naples Federico II, 80126 Naples, Italy

**Keywords:** Cancer genomics, Genome informatics, Cancer genomics

## Abstract

DNA methylation alterations are related to multiple molecular mechanisms. The DNA context of CpG sites plays a crucial role in the maintenance and stability of methylation patterns. The quantitative relationship between DNA composition and DNA methylation has been studied in normal as well as pathological conditions, showing that DNA methylation status is highly dependent on the local sequence context. In this work, we describe this relationship by analyzing the DNA sequence context associated to methylation profiles in both physiological and pathological conditions. In particular, we used DNA motifs to describe methylation stability patterns in normal tissues and aberrant methylation events in cancer lesions. In this manuscript, we show how different groups of DNA sequences can be related to specific epigenetic events, across normal and cancer tissues, and provide a thorough structural and functional characterization of these sequences.

## Introduction

Sizable efforts have characterized epigenetic signatures and their localization across the human genome^1^. However, the mechanisms influencing the sensitivity of certain sites to epigenetic modulation are still not completely understood as many internal and external factors are thought to be involved in this process. This aspect has been extensively studied in bacteria, where particular sequences are able to favor the binding of enzymes that modify the methylation status^2^.

It is well known that a dependency exists between DNA-sequence composition and occurrence of methylation in mammalian genomes. A well-known example of DNA-sequence-dependent mechanism is the dependency of DNA methylation on CpGs density^3,4^. Indeed, high density CpG clusters (typically CG Islands - CGIs) are usually located in the proximity of Transcription Start Sites (TSS) and promoters of housekeeping genes in an unmethylated state, hence allowing gene transcription^5,6^. Furthermore, a model of collaborative methylation among CpG sites has been recently suggested, explaining how CpGs in CGIs could maintain a stable methylation through the generations due to their clustered localization^7^. In 2009, McCabe and colleagues identified distinct sequence patterns marking methylation deregulation events in cancer^8^.

To date, only a few correlations between phenotypic alterations, DNA methylation alterations and DNA sequence context have been described^9,10^. Ghorbani and colleagues^11^ gave an insight to the identification of the motifs underlying common methylation patterns across different cancer types. Their results paved the way to the identification of alternative cancer biomarkers, currently needed in order to expand and diversify the therapeutic possibilities for cancer patients. In this context, epigenetic marks are considered as pivotal elements in biomarker discovery. For example, epigenetic signatures are used as diagnostic, prognostic and therapy-assessment markers in colorectal cancer^12^. However, the genomic context of aberrant methylated CpGs in cancer is still largely unexplored, and it remains unclear whether these contexts can be exploited to track tumor progression.

In this study, we analyzed DNA sequence contexts in the form of DNA motifs characterizing: (i) interindividual CpG methylation variability in normal tissues and (ii) aberrant CpG methylation between normal and cancer tissues. By using such approach we revealed the existence of i) tissue specific motifs characterizing low and high population variability of CpG methylation in normal tissues and (ii) putative cancer-specific biomarkers, constituted by sequence motifs related to aberrant methylation across 33 cancer types. Furthermore, we investigated the regulatory consequences of aberrant methylation in selected genomic contexts, in order to give an insight on the mechanisms underlying the gene expression deregulation in the onset and progression of cancer. Our results suggest the existence of alternative biomarkers in human cancer and provide novel hints in understanding of the role of the DNA sequence context in genetic and epigenetic deregulation.

## Materials and Methods

### Data sources

To investigate the relationship between DNA context and CpG methylation stability, we retrieved pre-processed DNA methylation data by Illumina Infinium 450 k assays from The Cancer Genome Atlas (TCGA)^13^ repository and the Epic Italy cohort^14^. Supplementary Table S1 reports, for each tissue type, the TCGA tissue IDs and the GEO Accessions for EPIC blood samples. For each tissue, we computed the variance distribution among the samples and selected two sets of CpGs: stable CpGs (**sCpGs**), showing variance values below the 1^st^ percentile, and unstable CpGs (**uCpGs**), showing variance above the 99^th^ percentile.

For the tumor samples, we collected CpGs from the dataset of informative differentially methylated CpGs (iDMCs) computed in^15^. Supplementary Table S3 (Supplementary File 1) reports the TCGA tissue IDs for each analyzed cancer type. This dataset was created starting from TCGA Illumina Infinium 450K assays. In particular, the authors selected CpGs whose methylation value was significantly different in the cancer tissue when compared to the normal counterpart or to a reference normal when the counterpart was not available. For each tissue the authors then selected the top 1000 differentially methylated CpGs having a variance value higher of a predefined threshold in cancer^16^. For each considered cancer type, we further divided these sets in hyper-methylated iDMC (hyper-iDMC) and hypo-methylated iDMC (hypo-iDMC) based on the observed trend of differential methylation^15^.

### Motif discovery

Given a set of CpGs of interest (normal or iDMCs) and the whole set of CpGs probed in the Illumina Infinium 450K platform as a background set, we searched for enriched motifs by using the Discriminative Regular Expression Motif Elicitation (*DREME*) tool from the Multiple EM for Motif Elicitation (*MEME)* suite^17^. In particular, for each CpG set, we provided *DREME* with two fasta files containing the *hg19* 20 bp flanking sequence of the CpGs in the set of interest and of the CpGs in the background set, respectively.

From the *DREME* output, we selected only those motifs with an E-value, i.e. the enrichment Fisher p-value corrected for multiple test on the number of candidate motifs, lower than 0.05.

Where the information on differential methylation was available, we classified a motif as mostly hyper- (hypo-) methylated if more than 70% of the CpGs carrying the motif were hyper- (hypo-) methylated, in all other cases the motif methylation status was considered as undefined. Moreover, for each motif in each tissue, we performed a hypergeometric test to evaluate the enrichment of CpGs carrying the motif for hyper- or hypo-methylation with respect to the corresponding background set. We considered as significantly hyper- or hypo-methylated the motifs with hypergeometric p-value lower than 0.05.

### Motifs genomic annotation

For each discovered motif, we selected the set of CpGs containing the motif in their surrounding sequence. We then annotated each set with *hg19* genes information, using the *IlluminaHumanMethylation450kanno*.*ilmn12*.*hg19* R package. In particular, we counted the number of CpGs in the set related to gene regions (1^st^Exon, 3′_UTR, 5′_UTR, Body, TSS1500, TSS200) and CGI regions (Island, N_Shelf, N_Shore, OpenSea, S_Shelf, S_Shore).

### Motifs association with transcription factors

In order to assess whether the discovered motifs encompass a Transcription Factor Binding Site (TFBS), we employed the *Tomtom* tool^18^ from the *MEME* suite. For each motif, we used *Tomtom* to search against the JASPAR 2018 (*JASPAR2018_CORE_vertebrates_non_redundant)* sequence database provided by the *MEME* suite. We considered for subsequent analyses only Jaspar TFBS having at least 5 bp overlap with the motif sequence and an E-value lower than 10.

### TF binding methylation affinity and expression

For each motif we considered the set of TFs, derived from the *Tomtom* tool, showing binding affinity. We then considered only the transcription factors (TFs) annotated as MethylPlus or MethylMinus in^19^. For each TF, we retrieved the list of potential target genes using the TRRUST database^20^. For each motif/TF pair, we retrieved the expression profiles of the TF target genes in the associated tumor and normal tissues using the FirebrowseR package^16^. We then performed a test for differential expression using a Wilcoxon test and defined each gene upregulated or downregulated in cancer, if the difference in median expression values between tumor and control was positive or negative and the Wilcoxon p-value was lower than 0.05. After that, we annotated the motif/TF pairs as associated with up-regulation if more than 50% of differentially expressed target genes were up-regulated, down-regulation if more than 50% of differentially expressed target genes were down-regulated, mixed-regulated otherwise.

## Results

### Sequence context as a marker of methylation instability in normal tissues

To investigate the relationship between DNA context and CpG methylation stability, we built a set of normal methylomes using DNA methylation data of normal tissues associated to 11 cancer types from TCGA and normal blood samples from the EPIC Italy cohort. Considering the variance distribution, we selected two sets of CpGs for each tissue: stable CpGs (**sCpGs**) and unstable CpGs (**uCpGs**).

For each sample, we considered in turn the set of selected stable and unstable CpGs and looked for recurrent DNA motifs (not necessarily including the CpG site) in the 20 bp flanking regions surrounding each CpGs and we found 108 motifs characterizing sCpGs and uCpGs in different tissues corresponding to overall 77 unique motifs (Supplementary Table S2). Out of these, 42 motifs were generated from uCpGs and 65 from sCpGs, with size ranging from 3 to 8 nucleotides (Supplementary Fig. S1).

**Figure 1:**
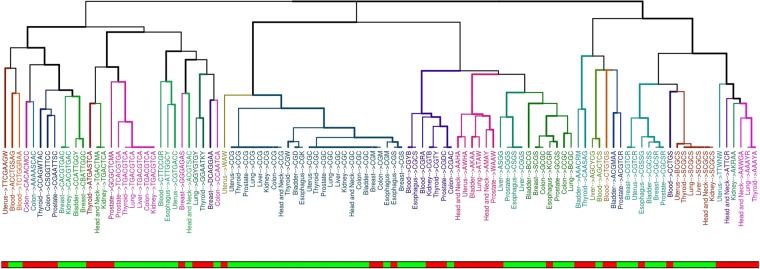
Clustering of motifs among the different normal tissues. For each motif the normal tissue and the sequence are reported. Labels’ color represents groups of similar motifs based on clustering. The bottom bar indicates the stability of the obtained motifs. sCpGs are shown in green while uCpGs in red.

We then compared the discovered motifs among the different tissues by means of a distance function based on sequence similarity and grouped them using hierarchical clustering (Fig. 1).

Interestingly, we found distinct patterns of clustering among stable and unstable sequence motifs. By doing so, we were able to identify at least 9 groups of highly similar motifs that were recurrently found in more than 3 tissues.

**Figure 2:**
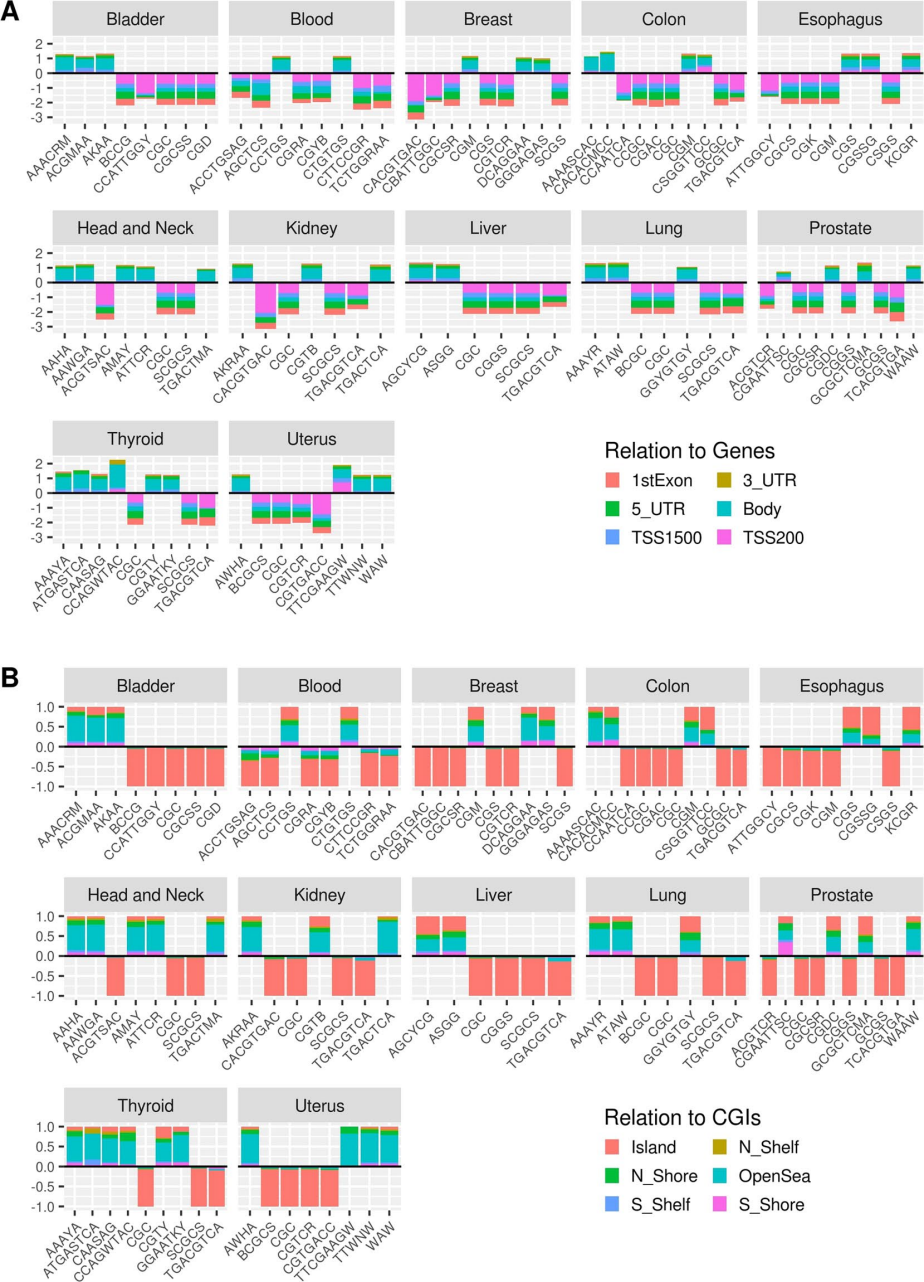
Genomic distribution of discovered motifs in normal tissues. Bar plots representing, for each analyzed tissue, the genomic distribution relative to gene regions (upper panel A) and CpG islands (lower panel B) respectively for motifs derived from uCpGs (positive values) and sCpGs (negative values).

Next, we characterized the relative positioning of motifs surrounding the stable and unstable CpGs by considering their localization with respect to genes (Fig. 2A) and to CpG dense regions (Fig. 2B). The sCpGs motifs are predominantly localized within the 5′ UTR and the 1^st^ exon, while the uCpGs motifs are mainly localized within the gene body (Fig. 2A). Interestingly, although uCpGs motifs are primarily located within the openSea regions, they maintain a discrete distribution in other genomic locations (Fig. 2B). sCpGs motifs were mainly localized within the proximal gene regulatory regions as well as islands.

### Sequence context as a marker of aberration in cancers

**Figure 3:**
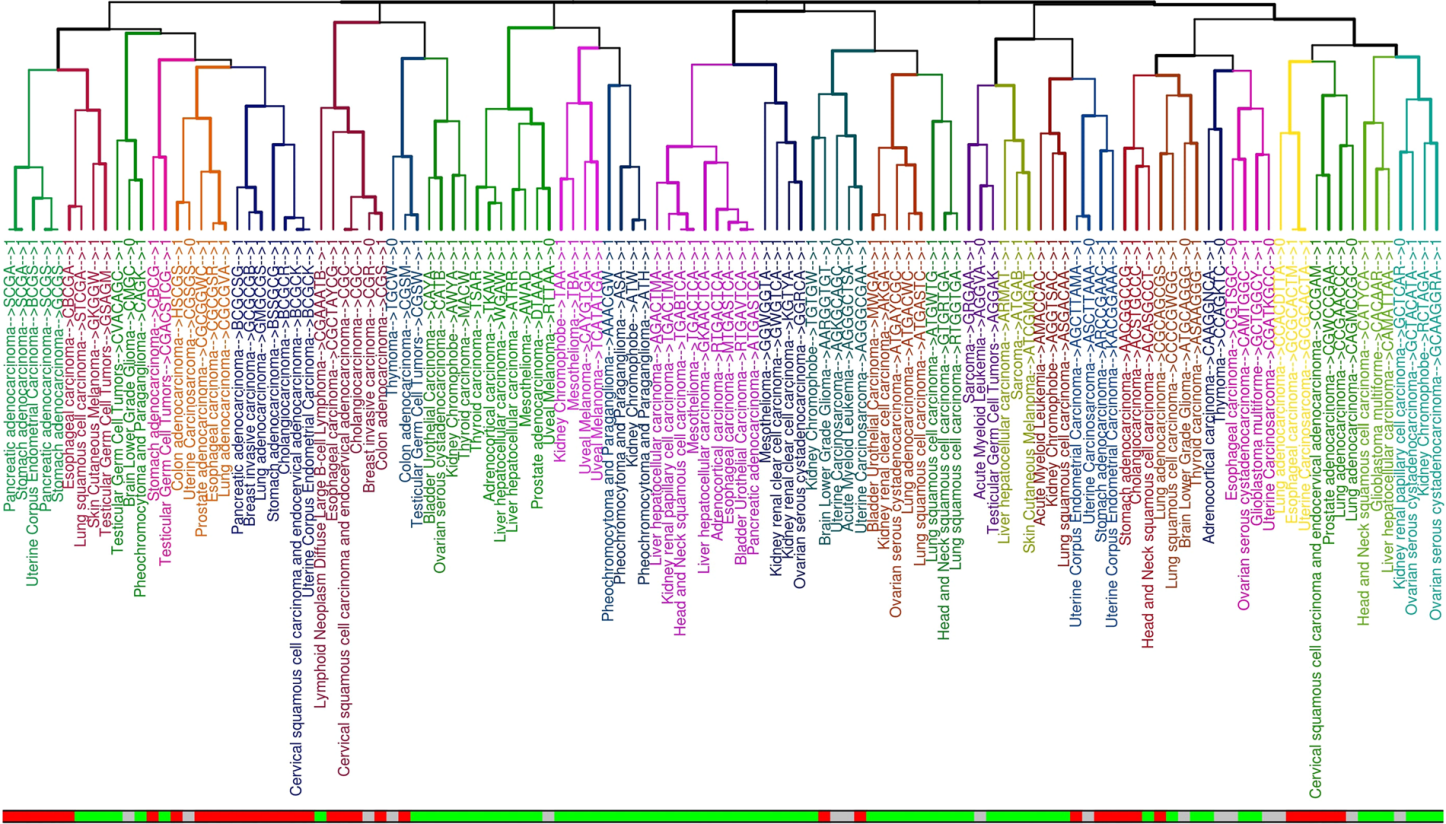
Clustering of motifs among different cancer tissues. For each motif the cancer tissue and the sequence are reported. The bottom bar indicates hypo-methylated motifs in green, hyper-methylated motifs in red, with undefined methylation status in grey. Labels’ color represents groups of similar motifs based on clustering.

We then considered 32 cancers from the TCGA (Supplementary Table S3) and used the dataset of informative differentially methylated CpGs (iDMCs) defined in^15^. We divided these sets, for each cancer type, in hyper-methylated (hyper-iDMC) and hypo-methylated iDMC (hypo-iDMC) based on their methylation trend in tumor with respect to normal. By following a similar approach as in the previous analysis we considered, for each cancer type, the entire set of iDMCs and looked for recurrent DNA motifs (not necessarily including the CpG sites) in the 20 bps flanking regions surrounding each iDMC and we discovered statistically overrepresented DNA motifs in almost all cancer types (Fig. 3, Supplementary Table S4). In particular, we found 120 motifs overall the considered tissues, corresponding to 114 unique motifs, with different sizes ranging from 3 to 8 nucleotides (Supplementary Fig. S2 and Table S4).

For each motif, we assigned a dominant methylation direction in cancer (Fig. 3, bottom bar). Considering the reported methylation status of the iDMCs carrying the motif, motifs were labeled as “hyper-methylated”, “hypo-methylated” and “undefined” (see Methods section). This analysis led to 68 hypo-methylated, 36 hyper-methylated and 16 undefined motifs.

Moreover, we characterized the motifs to be significantly hyper- or hypo-methylated if the CpGs underlying the definition of the motif were significantly enriched (Hypergeometric test, p < 0.05), given the background composition of input iDMCs, for the particular aberration (Supplementary Table S2).

In order to ascertain how similar signatures were distributed among different cancer types and different aberration types (hypo- or hyper-methylation), we computed a distance function based on the sequence similarity among all pairs of derived motifs and performed a hierarchical clustering (Supplementary Fig. S3).

**Figure 4:**
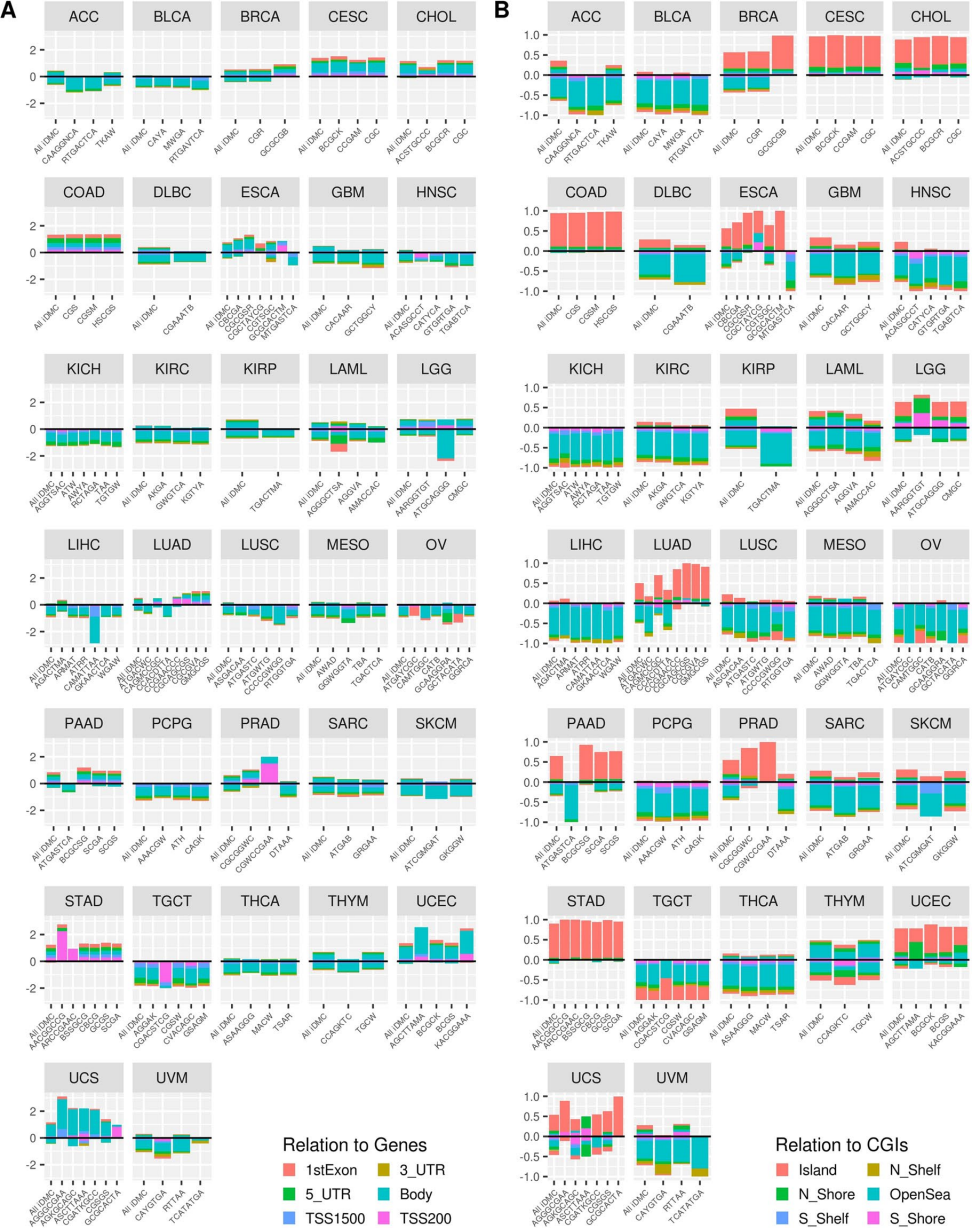
Genomic distribution of cancer methylation motifs. Bar plots representing, for each analyzed cancer tissue, the genomic distribution relative to gene regions (left panel A) and CpG islands (right panel B) respectively for motifs derived from hyper-iDMC (positive values) and hypo-iDMC (negative values).

Moreover, we analyzed the localization of the motifs associated with aberrant methylation in cancer samples (Fig. 4). We observed a neat positioning pattern of hypermethylated motifs within CG dense regions (Fig. 4B). In fact, in BRCA, CESC, CHOL, COAD, ESCA, LGG, LUAD, PAAD, PRAD, STAD, UCEC and UCS, the hypermethylated motifs are mainly located within CG-rich regions as well as CpG islands, while a marked localization is visible within the shore regions in LGG and in UCEC. On the other hand, the hypomethylated motifs are located mostly in openSea regions. Concerning protein coding genes, we observed a predominance of hypomethylated regions falling within the gene bodies (Fig. 4A).

### Binding site affinity in cancer motifs

**Figure 5:**
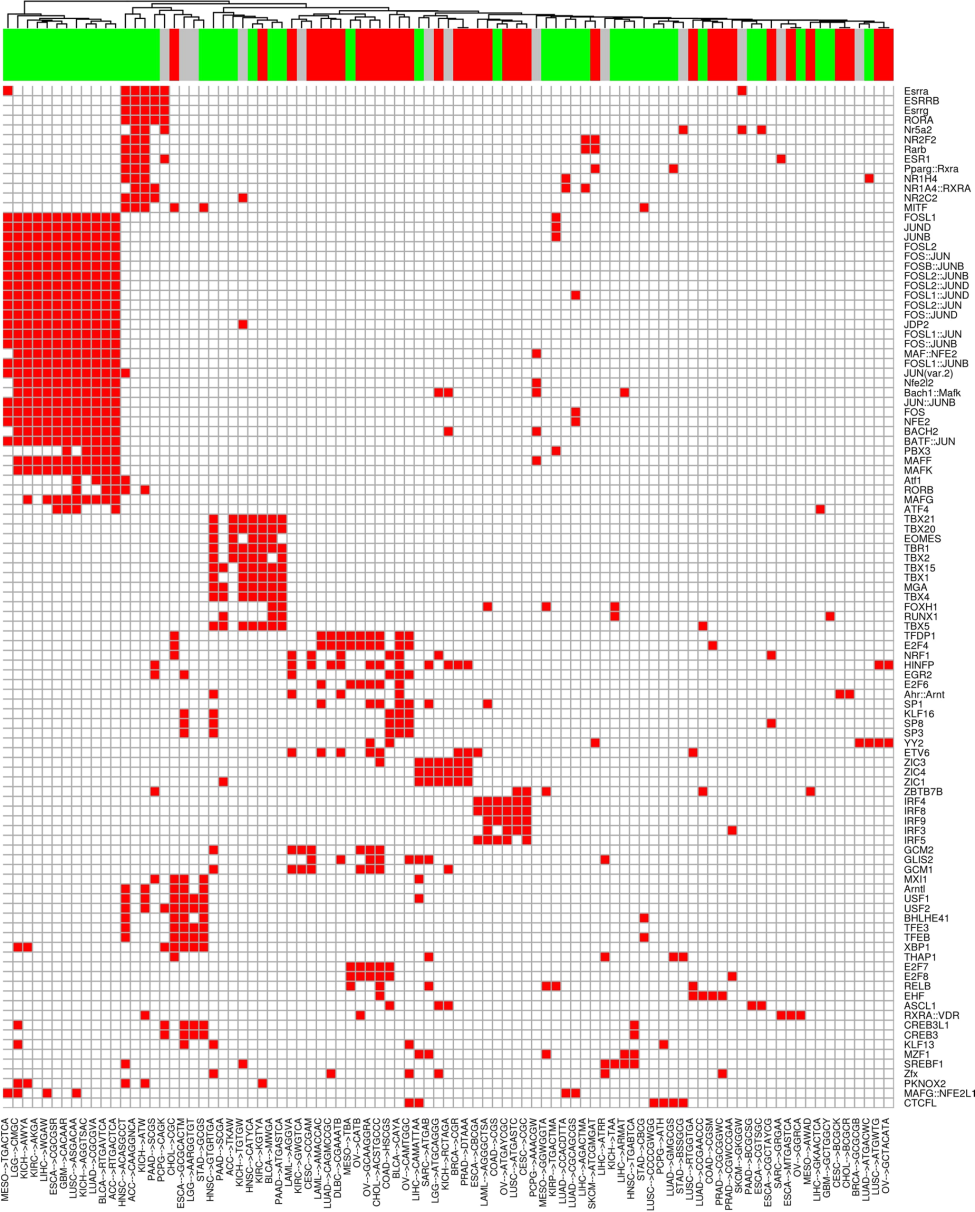
Transcription factors frequently associated with motifs. Heatmap reporting transcription factors whose binding sites are associated with motifs in at least 4 different tissues. Columns report each motif/cancer pair while rows report enriched TFs. Motifs are clustered based on the number of shared enriched TFs. The upper bar indicates hypo-methylated motifs in green, hyper-methylated motifs in red, with undefined methylation status in grey.

In order to investigate how the methylation status of enriched motifs can affect the transcriptional regulation of their potential target genes in cancer, we looked for transcription factors (TFs) whose binding domains have a potential affinity for the discovered motifs. Our analysis highlighted that most enriched transcription factors are both related to basal transcriptional regulation as well as to epigenetic mechanisms (Fig. 5). For instance, the Fos-Jun complex is the most enriched overall, followed by the Histone H4 nuclear factor P (HINFP). Similarly, we identified multiple motifs with potential binding affinity for the E2F transcription factors.

To better characterize the regulatory role of the motifs in the malignant phenotype, we identified transcriptional regulators acting both in a specific cancer and across multiple types of cancers. As shown in Fig. 5, a plethora of motifs share affinity for common transcriptional regulators (even in different cancers), mainly involved in basal transcriptional regulation machinery, such as the above mentioned Fos-Jun heterodimer and their paralogue proteins, Bach and Maf.

Such regulators were enriched in several cancer types, as well as lung, bladder cancer and kidney renal clear cell carcinoma. On the other hand, a distinct regulatory signature sustained by the IRF family regulators shared by stomach, cervical squamous cell carcinoma and in lymphoid neoplasm diffuse large B-cell lymphoma emerged. Furthermore, our findings show a specific enrichment of the ETV family regulators in colon adenocarcinoma and uterine corpus endometrial carcinoma. While such patterns of regulation are peculiar for the mentioned cancers, we observed a remarkably complex regulation in lung and prostate adenocarcinoma as well as in esophageal cancer.

### The methylation status of hyper-iDMC and hypo-iDMC affects gene expression through TFs binding

Recently, different affinity binding of TFs to their target sites based on their methylation status has been thoroughly characterized^19^. In order to strengthen our findings from a mechanistic point of view, we tested the hypothesis that there is a concordance between the methylation status of the considered motifs, the methylation-dependent binding affinity of the transcriptional regulators, and the expression trend of their target genes. To achieve this goal, we first classified each association TF/motif as concordant if the TF was reported as MethylPlus in^19^ and associated to a hyper-methylated motif or if it was classified as MethylMinus and associated to a hypo-methylated motif in the corresponding cancer tissue, as discordant otherwise (Supplementary Table S5).

Summarizing over all cancer tissues, we found that when considering our motifs as potential binding sites, the concordance was confirmed only for MethylMinus TFs.

Then, we linked this information with differential expression of available cancer tissues and we found that a notable group of concordant TFs target genes show expression patterns in line with the methylation status of the motif and the TFs preference for methylated or unmethylated targets (Table 1).

**Table 1:** Number of identified target genes showing only down-regulation, mixed regulation and up-regulation patterns among cancer tissues.

	Down-regulation	Mixed-regulation	Up-regulation
Concordant TFs	5	0	24
Discordant TFs	4	5	3

Differentially expressed target genes among all cancer tissues are divided on the base of the concordance of their targeting TFs methylation affinity and their TFBS methylation status. We define as “Concordant TFs” the TFs classified as MethylPlus and associated to a hyper-methylated motif or classified as MethylMinus and associated to a hypo-methylated motif in the corresponding cancer tissue. On the contrary, we define as “Discordant TFs” the TFs classified as MethylMinus and associated to a hyper-methylated motif or classified as MethylPlus and associated to a hypo-methylated motif in the corresponding cancer tissue.

## Discussion

In this work, we explored the relationship between CpG methylation changes and the presence of recurring DNA sequences in the surrounding region of CpG sites. The analysis has been conducted on CpGs showing different methylation variation patterns in normal tissues and CpGs associated with aberrant methylation in cancer tissues.

In normal tissues, all of the motifs that were found enriched in more than one tissue, were predominantly associated to the same stability pattern. This suggests that their association with methylation stability is conserved across different tissues. Short motifs were mostly composed by a CpG dinucleotide followed by a further C or G, most probably indicating the origin of the motif in a CpG-rich region.

Interestingly, structures formed by more than 4 nucleotides, such as the *TGACGTCA* motif, were retrieved in their exact sequence in prostate, thyroid, lung, liver, colon and kidney. Notably, the CpG methylation status of such motif has been extensively studied. In fact, *TGACGTCA* has been identified as a pivotal factor in regulating cell type- and development stage-specific gene expression^21^. The *TGACGTCA* motif (namely the cAMP-responsive enhancer/promoter sequence) is specifically occurring upstream of genes whose expression is regulated by cyclic adenosine monophosphate molecules, and its aberrant methylation is related to the loss of binding properties by ATF family regulators and subsequent transcriptional inactivation.

Moreover, we found the *CACGTGAC* motif significantly overrepresented in breast and kidney. Anantharaman and colleagues^22^ investigated the cis-regulatory role of this motif in the basal regulation of transcription highlighting the capability of such motif to bind HLH proteins in erythroid cell line differentiation. In this context, the *CACGTGAC* motif is known as E-box. Furthermore, early *in vitro* studies demonstrated that this motif constitutes the binding site for the transcription factors USF1 and USF2^23,24^. These transcriptional regulators can act both as homo or heterodimer recruiting the TFIID complex to the promoter. This motif has been already described as having a high affinity binding for multiple other transcription factors such as NF-E2, GATA-1, EKLF, and Tal1, *in vivo*^25^.

The *CCAATCA* motif, enriched in the colon tissue, is localized in the region surrounding one of the most diffused cis-regulators in the basal transcription machinery, the CCAAT box. Meergans and colleagues^26^ clarified the role of this well conserved sequence in the expression regulation of genes coding for the histone proteins H1, the main factor guiding the nucleosome positioning. Interestingly, the so-called CCAAT box is highly conserved in all the promoters of the H1 loci and it is followed by a CA dinucleotide.

A correlation between the local methylation profiles and CpGs density is known^27–29^. CpGs proximal to a TSS show less variation (more stability) than distal CpGs^6^. In this context, our results support the hypothesis that stable CpG motifs are related to high CpG content and located in promoter regions, while unstable CpG motifs are related to low CpG content and located in gene body regions.

Similarly, in Acute Lymphoid Leukemia cells it has been shown that CpGs located outside the CGIs show a higher variation (instability) than those falling within the CGIs^30^.

Methylation changes occur not only during physiological processes but also in several pathological conditions including cancer^25^. To date, the exact mechanism that modulates the sensitivity of CpGs to methylation is still unknown. A seminal study by Feltus *et al*. reported that aberrant methylation relies on local sequence composition^31^.

Having observed the existence of motif structures associated with CpG methylation stability in normal tissues, we ascertained whether similar structures can be found when considering aberrant methylation associated with cancer tissues.

For instance, the *TGACTCA* motif, which was statistically overrepresented in mesothelioma and in its variant *TGACTMA* in kidney, has been already identified as the binding motif of the transcriptional complex AP1^32^. Seldeen *et al*.^32^, inspected the effect of constitutive modifications of the *TGACTCA* motif, given the important role of the Fos-Jun transcription factors in the translation of extracellular signals in gene expression regulation (by growth factors and cytokines).

In order to disentangle the motif relatedness among different tissues from the similarity of the corresponding CpG input sets, we built a dendrogram based on the Jaccard distance among sets of iDMC for each tumor (Supplementary Fig. S3) and observed a different clustering of cancers, mainly driven by their tissue of origin. This suggests that tissue similarities found by considering motifs structure are not only dependent on the amount of shared iDMCs, but also by the structure composition of enriched motifs generated by different sets of CpG sites. When considering the methylation direction, we observed that the clustering of structurally similar motifs is consistent with the direction of methylation change. Notably, we observed that motifs which are prone to have a hypermethylated profile are GC-rich in respect to the other ones. This phenomenon has been previously described and reported in^33^ as CpG island methylator phenotype (CIMP). Although CIMP has been reported in several cancers, including gastric^34–37^, lung^38^, liver^39^, ovarian^40^, endometrial^41^, breast^42^, gliomas^43^ and leukemias^44^, most of the literature relates its effects to colorectal cancers (CRC)^45^. Currently, the CIMP causative factors are still unknown^46^. However, the investigation of CIMP in different cancers led to the hypothesis of an epigenetic-driven onset of the malignant transformation, in turn leading to the inactivation of essential genes as well as tumor suppressors. The results obtained in the present work are also in line with this hypothesis. When looking at the genomic localization, we found that enriched motifs in STAD and PRAD are mainly localized within the 200 bp sequence surrounding the transcription start site, suggesting their possible direct involvement on the corresponding gene expression activity. Once obtained a list of motifs associated with each cancer type, we investigated their potential transcription factor binding affinity.

For instance, the motifs encompassing the HINFP binding sites, are among the most enriched overall the considered cancers. HINFP is a crucial regulator of the expression of genes encoding the H4 histones. Therefore, its function is essential in the S phase of the cell cycle, affecting the packaging of newly synthetized DNA into condensed chromatin^47^. This raises the perspective that aberrant methylation in motifs surrounding the HINFP binding sites could be causative of genomic instability and malignant transformation. On the other hand, our results suggest a possible involvement of the discovered motifs in the regulation of genes targeting the AP-1 heterodimer. This is also supported by the presence of motifs related to the NFE2 transcription factor, since this subunit is able to recognize the 5′-TGA(C/G)TCA-3′ sequence of the AP-1-like binding site^48^. This regulatory complex affects the expression of a wide range of genes. Dimerization of Fos-Jun proteins with members of the Maf and NF-E2 (CNC) families further expands the range of Fos-Jun targeting. In addition, different Jun and Fos family members can have opposite effects on AP-1-mediated transactivation^49–51^. Notably, by looking at the methylation status of the considered motifs, we noticed that this cluster of transcriptional regulators is typically related to hypomethylated motifs.

Similarly, markers of E2F-dependent transcription are mostly represented overall cancers types, as well as E2F4 and TFDP1, which regulate genes involved in key processes of malignant transformation, such as cell cycle regulation and DNA replication^52,53^.

Deregulation of E2F-dependent G1/S transcription (i.e by certain oncogenes), leads the cells to enter the S-phase, triggering cell proliferation^54^. For this reason, we suppose that hypermethylation at E2F binding motifs, which we found overrepresented in colon and stomach adenocarcinoma as well as in sarcoma, could lead to an unmodulated E2F-dependent transcription of targets involved in the G1/S checkpoint and, thus, uncontrolled cell proliferation. Our results show that the TFs binding affinity for motifs marking aberrant DNA methylation regions involves both Fos-Jun regulators (as found in most of the cancers), and other specific factors, shared among few cancers, suggesting a possible crucial role of these motifs in the development of cancer phenotypes. On the opposite side, we identified motifs bound by a single transcription factor. In this case, the motif CGC, found overrepresented in cholangiocarcinoma, cervical squamous cell carcinoma and endocervical adenocarcinoma, is predicted to be bound specifically by Ahr::Arnt. Similarly, the motifs CGR (breast invasive carcinoma), CACAAR (glioblastoma multiforme), ATGWTG (lung squamous cell carcinoma), CGWCCGAA (prostate adenocarcinoma) show exclusive affinity for YY2, RUNX1, ATF4 and ZBTB7B, respectively.

We finally checked the concordance among the methylation status of the observed motifs, the TFs binding affinity for methylated or unmethylated sites and TFs target genes expression deregulation. The observed results suggest that the identified DNA methylation motifs not only represent potential biomarkers, but they contribute to disentangle the complex regulatory circuits in cancer genomes.

## Conclusions

The relationship between sequence context and DNA methylation is an important feature to gain further insights on the dynamics of methylation establishment, maintenance and alteration in pathological conditions. Here, we provided the first comprehensive catalogue of short DNA sequences that can be associated to methylation stability between individuals in normal tissues and to aberrant methylation conditions in different cancer types. We showed how different DNA contexts could predispose a CpG to be more or less prone in gaining or losing methyl groups. We characterized the genomic localization of these sequences and their relationship with transcriptional regulators in the genome. When considering motifs associated to aberrant CpGs characterizing cancer tissues, we showed that some motifs are shared by different cancer types while others are cancer specific. This latter aspect could be exploited to define more precise and effective molecular targets in gene-based therapies such as those employing epigenetic CRISPR-Cas9 strategies.

## Supplementary information

Supplementary Information.
