# Supplementary material for: DNA sequence context as a marker of CpG methylation instability in normal and cancer tissues
Source: Sci Rep. 2020 Feb 3;10:1721. doi: 10.1038/s41598-020-58331-w (PMC6997448; doi:10.1038/s41598-020-58331-w)
Supplement: Supplementary file 1 — Supplementary Information. [file 41598_2020_58331_MOESM1_ESM.pdf]

DNA sequence context as a marker of CpG methylation instability in normal and cancer tissues

Giovanni Scala[1], Antonio Federico[1], Domenico Palumbo[2], Sergio Cocozza[2], Dario Greco[1,3,4,*]

1. Faculty of Medicine and Health Technology, Tampere University, Finland;
2. Department of Molecular Medicine and Medical Biotechnology, University of Naples "Federico II", Italy;
3. BioMediTech Institute, Tampere University, Finland;
4. Institute of Biotechnology, University of Helsinki, Finland.

* Corresponding author: dario.greco@tuni.fi

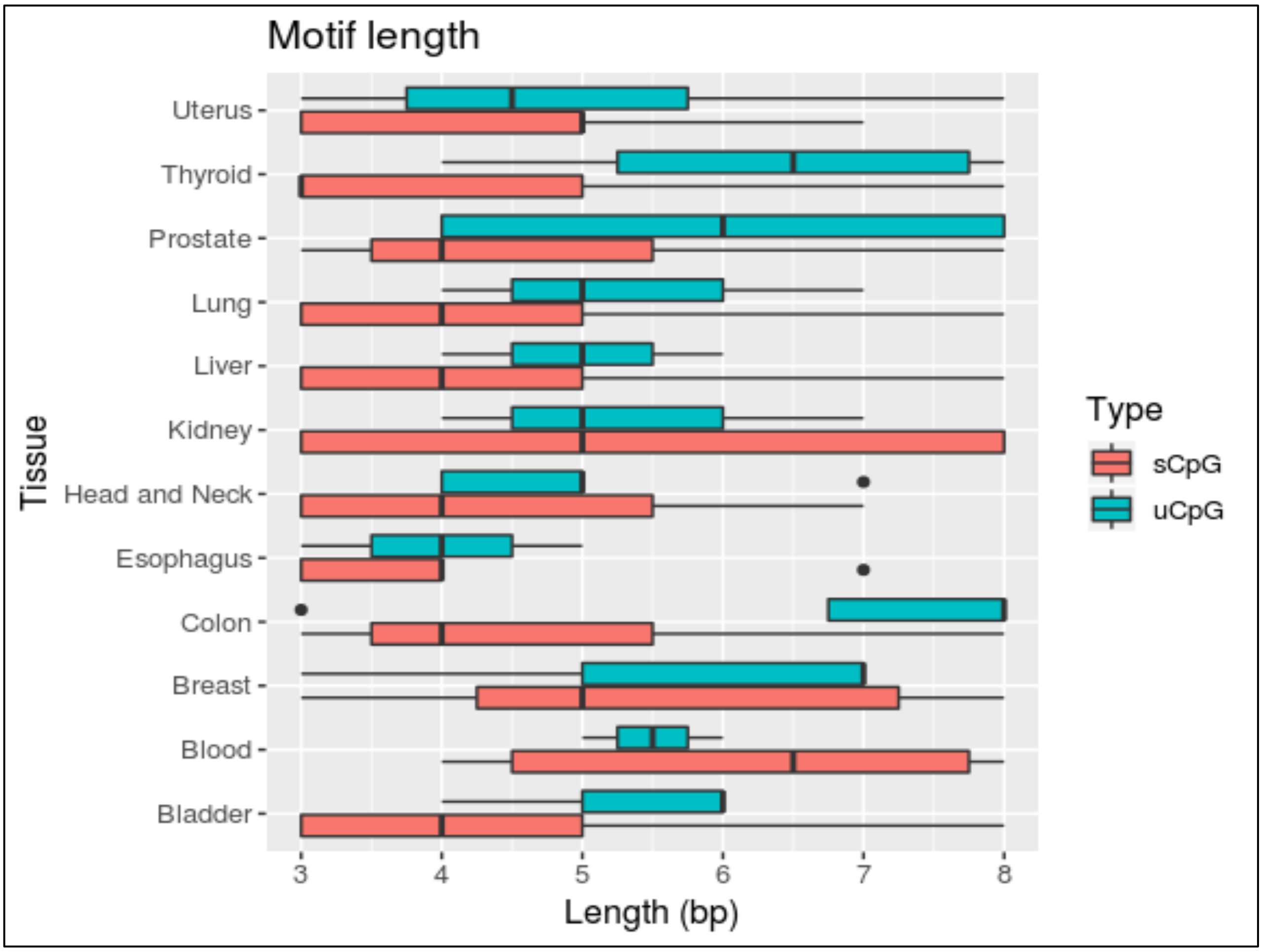

Figure S1. Distribution sCpG (blue) and uCpG (red) motif sizes for each normal tissue.

We compared the discovered motif among the different tissues by computing a distance function based on their sequence similarity and grouped them using hierarchical clustering (Figure 1).

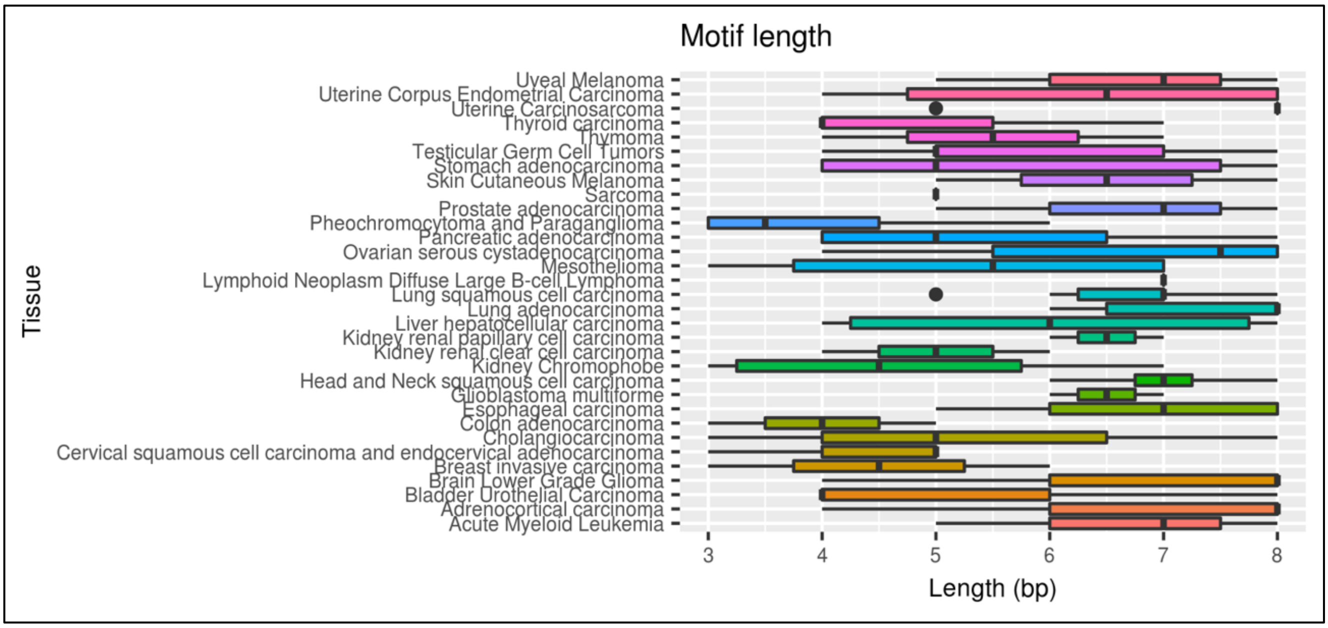

Figure S2. Distribution of motifs length for each cancer type.

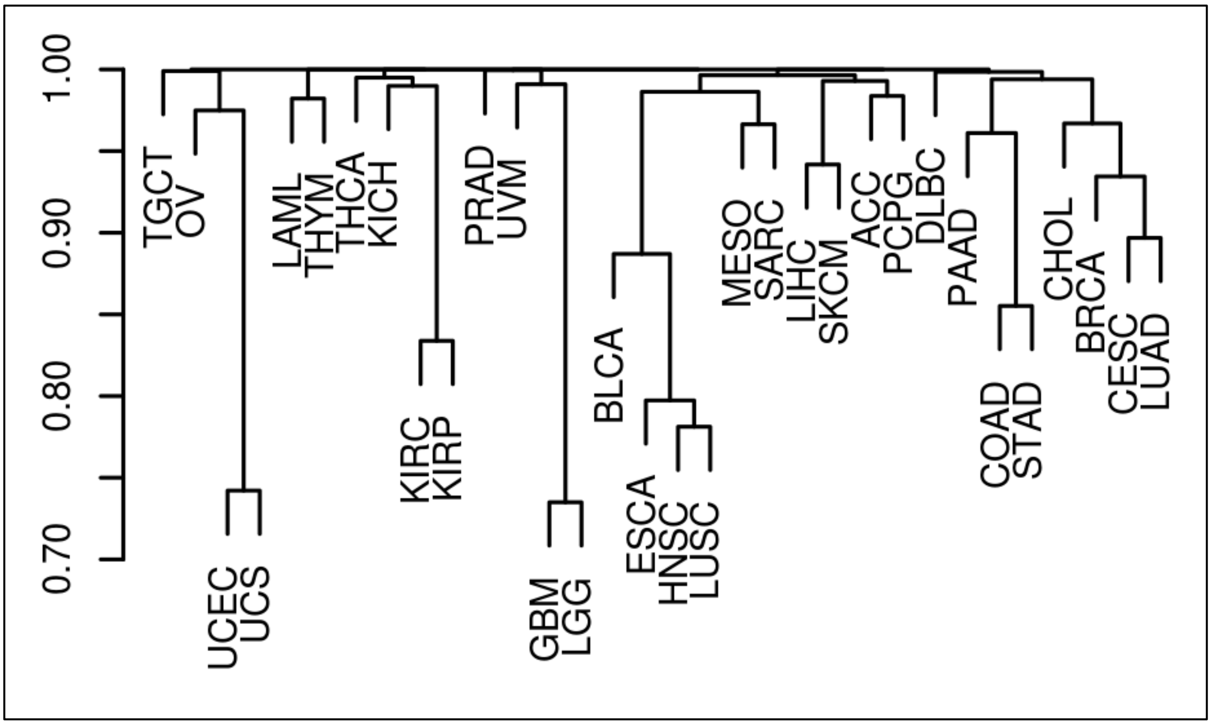

Figure S3. Clustering of the different cancer tissues based on the iDMC set similarity. The dendrogram reports the set similarity of the original input sets. Pairwise similarity is computed as the complement to one of the Jaccard similarity.

Table S1. Normal tissues considered in the study

CODE	Tissue	Dataset
BLCA	Bladder	TCGA
EPIC	Blood	EPIC ITALY
BRCA	Breast	TCGA
COAD	Colon	TCGA
ESCA	Esophagus	TCGA
HNSC	Head and Neck	TCGA
KIRC	Kidney	TCGA
LIHC	Liver	TCGA
LUSC	Lung	TCGA
PRAD	Prostate	TCGA
THCA	Thyroid	TCGA
UCEC	Uterus	TCGA

Table S2. DNA motifs associated with SCpGs and uCpGs in normal tissues. Columns legend: **Variance** - uCpGs (unstable CpGs) and sCpGs (stable CpGs); **Motif** - the sequence motif identified; **Consensus Sequence** - motif consensus sequences; **Cluster -** motif cluster id; **Length** - length of the identified motif; **Positive** - number of CpGs with the corresponding motif in the input set; **Total positive** - number of total CpGs in the input set; **Negative** - number of CpGs with the corresponding motif in the background set; **Total negative** - number of total CpGs in the background set; **Positive ratio** - ratio between Positive and Total positive; **Negative ratio** - ratio between Negative and Total negative; **p-value** - motif enrichment P-value; **e-value** - motif enrichment e-value.

	A	B	C	D	E	F	G	H	I	J	K	L	M	N	O
1	**Tissue ID**	**Tissue**	**Variance**	**Motif**	**Consensus Sequence**	**Cluster**	**Length**	**Positive**	**Total positive**	**Negative**	**Total negative**	**Positive ratio**	**Negative ratio**	**p-value**	**e-value**
2	PRAD	Prostate	uCpGs	GCGCTCMA	**GCGCTCMA**	23	8	29	3369	1404	485512	0,008607896	0,002891793	0,0000005	0,015
3	THCA	Thyroid	uCpGs	ATGASTCA		19	8	23	3401	865	485512	0,006762717	0,001781624	0,00000014	0,0053
4	PRAD	Prostate	sCpGs	TCACGTGA	**TCACGTGA**	29	8	15	3369	473	485512	0,00445236	0,000974229	0,0000023	0,046
5	THCA	Thyroid	sCpGs	TGACGTCA		29	8	32	3399	741	485512	0,009414534	0,001526224	2,8E-15	6E-11
6	LUSC	Lung2	sCpGs	TGACGTCA		29	8	31	3403	741	485512	0,009109609	0,001526224	1,8E-14	3,5E-10
7	LIHC	Liver	sCpGs	TGACGTCA		29	8	22	3336	741	485512	0,006594724	0,001526224	0,000000029	0,00058
8	COAD	Colon	sCpGs	TGACGTCA		29	8	25	3343	741	485512	0,007478313	0,001526224	3E-10	0,0000061
9	KIRC	Kidney2	sCpGs	TGACGTCA		29	8	28	3297	741	485512	0,008492569	0,001526224	1,5E-12	0,000000031
10	UCEC	Uterus2	uCpGs	TTCGAAGW	**TTCGAAGW**	26	8	10	3374	184	485512	0,002963841	0,000378981	0,0000013	0,027
11	EPIC	Blood3	sCpGs	ACCTGSAG	**ACCTGSAG**	32	8	36	2065	2482	485512	0,017433414	0,005112129	6,7E-10	0,000017
12	EPIC	Blood3	sCpGs	TCTGGRAA	**TCTGGRAA**	33	8	27	2065	1949	485512	0,013075061	0,004014319	0,00000021	0,005
13	COAD	Colon	uCpGs	AAAASCAC	**AAWASCRS**	9	8	25	3341	1157	485512	0,00748279	0,002383051	0,0000012	0,034
14	COAD	Colon	uCpGs	CACACMCC	**CACACMCC**	7	8	39	3341	2116	485512	0,011673152	0,004358286	0,000000089	0,0026
15	BRCA	Breast	sCpGs	CACGTGAC	**CACGTGAC**	28	8	21	3402	865	485512	0,00617284	0,001781624	0,0000019	0,039
16	KIRC	Kidney2	sCpGs	CACGTGAC		28	8	23	3297	964	485512	0,006976039	0,001985533	0,0000005	0,01
17	BLCA	Bladder	sCpGs	CCATTGGY	**CBATTGCY**	27	8	21	3425	824	485512	0,006131387	0,001697177	0,000001	0,018
18	BRCA	Breast	sCpGs	CBATTGGC		27	8	22	3402	844	485512	0,006466784	0,001738371	0,00000035	0,0072
19	THCA	Thyroid	uCpGs	CCAGWTAC	**CSRRWTVC**	8	8	19	3401	695	485512	0,005586592	0,001431479	0,0000011	0,038
20	COAD	Colon	uCpGs	CSGGTTCC		8	8	28	3341	1340	485512	0,008380724	0,002759973	0,00000053	0,016
21	PRAD	Prostate	uCpGs	CGAATTSC		8	8	16	3369	423	485512	0,004749184	0,000871245	0,00000011	0,0034
22	LUSC	Lung2	uCpGs	GGYGTGY	**GGHRTKY**	22	7	141	3385	11772	485512	0,041654357	0,024246569	1,5E-09	0,000059
23	THCA	Thyroid	uCpGs	GGAATKY		22	7	108	3401	7723	485512	0,031755366	0,015906919	5,7E-11	0,0000022
24	BRCA	Breast	uCpGs	GGGAGAS	**GGGAGAS**	6	7	109	3401	9103	485512	0,032049397	0,018749279	0,00000014	0,0039
25	HNSC	Head and N	sCpGs	ACGTSAC	**ACGTSAC**	36	7	65	3361	3918	485512	0,019339482	0,008069831	4,9E-10	0,0000099
26	BRCA	Breast	uCpGs	DCAGGAA	**DYAKDAA**	5	7	131	3401	10785	485512	0,038518083	0,022213663	3,4E-09	0,000099
27	COAD	Colon	sCpGs	CCAATCA	**CCAATCA**	30	7	37	3343	2016	485512	0,011067903	0,004152318	0,00000021	0,0041
28	HNSC	Head and N	uCpGs	TGACTMA	**TGACTMA**	14	7	68	3360	3094	485512	0,020238095	0,006372654	9,3E-16	3,3E-11
29	KIRC	Kidney2	uCpGs	TGACTCA		14	7	64	3297	2490	485512	0,019411586	0,005128607	2,2E-18	8,6E-14
30	EPIC	Blood3	sCpGs	CTTCCGR	**CTTCCGR**	31	7	71	2065	5864	485512	0,034382567	0,012077971	2,9E-14	7,5E-10
31	ESCA	Esophagus	sCpGs	ATTGGCY	**MKTGRCY**	34	7	54	3404	3298	485512	0,01586369	0,006792829	0,000000031	0,00057
32	UCEC	Uterus2	sCpGs	CGTGACC		34	7	26	3374	959	485512	0,007705987	0,001975234	0,000000013	0,00027
33	UCEC	Uterus2	uCpGs	WAW	**WAW**	25	3	1947	3374	243979	485512	0,57705987	0,50251899	3,1E-18	9E-14
34	UCEC	Uterus2	sCpGs	CCG	**CCG**	4	3	2200	3374	271269	485512	0,65204505	0,558727694	2,5E-28	6,5E-24
35	THCA	Thyroid	sCpGs	CCG		4	3	2237	3399	272622	485512	0,658134746	0,561514442	1,6E-30	4,1E-26
36	PRAD	Prostate	sCpGs	CCG		4	3	1040	3369	117540	485512	0,308696943	0,242094943	1,3E-18	2,9E-14
37	LUSC	Lung2	sCpGs	CCG		4	3	2255	3403	272622	485512	0,662650602	0,561514442	2,4E-33	5,9E-29
38	LIHC	Liver	sCpGs	CCG		4	3	957	3336	117770	485512	0,286870504	0,24256867	2,9E-09	0,000065
39	KIRC	Kidney2	sCpGs	CCG		4	3	2154	3297	272622	485512	0,653321201	0,561514442	6E-27	1,5E-22
40	COAD	Colon	sCpGs	CCG		4	3	2287	3343	278426	485512	0,684116063	0,573468833	2,2E-39	5,5E-35
41	HNSC	Head and N	sCpGs	CCG		4	3	2226	3361	272622	485512	0,662302886	0,561514442	9,4E-33	2,4E-28
42	THCA	Thyroid	sCpGs	CGW	**CGD**	4	3	1274	3399	156036	485512	0,374816122	0,321384435	3E-11	0,00000064
43	BLCA	Bladder	sCpGs	CGD		4	3	1636	3425	191999	485512	0,477664234	0,395456755	1,6E-22	3,6E-18
44	ESCA	Esophagus	sCpGs	CGK		4	3	1085	3404	113733	485512	0,318742656	0,234253736	2,8E-29	6E-25
45	UCEC	Uterus2	sCpGs	CGC	**CGC**	4	3	2410	3374	232134	485512	0,714285714	0,478122065	3,9E-169	1,3E-164
46	THCA	Thyroid	sCpGs	CGC		4	3	2611	3399	255170	485512	0,768167108	0,525568884	2E-186	6,8E-182
47	PRAD	Prostate	sCpGs	CGC		4	3	1561	3369	156251	485512	0,463342238	0,321827267	4,4E-65	1,3E-60
48	LUSC	Lung2	sCpGs	CGC		4	3	1214	3403	109457	485512	0,356744049	0,225446539	4E-67	1,1E-62
49	LIHC	Liver	sCpGs	CGC		4	3	2553	3336	255170	485512	0,76528777	0,525568884	1,6E-178	5,1E-174
50	KIRC	Kidney2	sCpGs	CGC		4	3	2528	3297	255170	485512	0,766757658	0,525568884	9,8E-179	3,1E-174
51	HNSC	Head and N	sCpGs	CGC		4	3	2574	3361	255170	485512	0,765843499	0,525568884	1,1E-180	3,4E-176
52	COAD	Colon	sCpGs	CGC		4	3	1922	3343	184095	485512	0,574932695	0,379177034	3,4E-115	1E-110
53	BLCA	Bladder	sCpGs	CGC		4	3	2641	3425	261601	485512	0,771094891	0,538814695	8,2E-174	2,8E-169
54	BRCA	Breast	uCpGs	CGM	**CGM**	4	3	3061	3401	415148	485512	0,900029403	0,855072583	4,1E-15	1,6E-10
55	COAD	Colon	uCpGs	CGM		4	3	2973	3341	415148	485512	0,889853337	0,855072583	2,1E-09	0,000086
56	ESCA	Esophagus	sCpGs	CGM		4	3	2196	3404	247572	485512	0,645123384	0,509919425	8,9E-57	2,5E-52
57	ESCA	Esophagus	uCpGs	CGS	**CGS**	4	3	2175	3397	284034	485512	0,640270827	0,585019526	3E-11	0,00000096
58	BRCA	Breast	sCpGs	CGS		4	3	2583	3402	305571	485512	0,759259259	0,629378882	8,3E-59	2,4E-54
59	EPIC	Blood3	sCpGs	CGYB		18	4	1887	2065	395677	485512	0,913801453	0,814968528	1,2E-36	4,4E-32
60	ESCA	Esophagus	sCpGs	CGCS		18	4	2689	3404	217650	485512	0,789952996	0,448289641	0	0
61	EPIC	Blood3	sCpGs	CGRA		18	4	553	2065	104019	485512	0,26779661	0,214245992	4,8E-09	0,00012
62	PRAD	Prostate	uCpGs	CGDC		18	4	2039	3369	271278	485512	0,605224102	0,558746231	0,00000003	0,001
63	COAD	Colon	sCpGs	CGAC		18	4	257	3343	26583	485512	0,076877057	0,054752509	0,000000067	0,0014

	A	B	C	D	E	F	G	H	I	J	K	L	M	N	O
64	KIRC	Kidney2	uCpGs	CGTB		18	4	1891	3297	257349	485512	0,573551714	0,53005693	0,00000032	0,012
65	THCA	Thyroid	uCpGs	CGTY		18	4	1324	3401	169118	485512	0,389297266	0,348329187	0,00000039	0,015
66	HNSC	Head and N	uCpGs	AAHA	**WNHH**	2	4	1700	3360	194685	485512	0,505952381	0,400989059	1,1E-34	4,6E-30
67	UCEC	Uterus2	uCpGs	AWHA		2	4	1624	3374	181359	485512	0,481327801	0,373541746	3,4E-37	1,2E-32
68	BLCA	Bladder	uCpGs	AKAA		2	4	1350	3401	171104	485512	0,396942076	0,352419714	0,000000043	0,0017
69	LUSC	Lung2	uCpGs	ATAW		2	4	536	3385	61228	485512	0,158345643	0,126110168	0,000000029	0,0011
70	HNSC	Head and N	uCpGs	AMAY		2	4	1659	3360	210544	485512	0,49375	0,433653545	1,7E-12	0,00000006
71	PRAD	Prostate	uCpGs	WAAW		2	4	1561	3369	193818	485512	0,463342238	0,399203315	3,1E-14	1,3E-09
72	LIHC	Liver	uCpGs	ASGG	**MSGS**	21	4	1893	3332	255262	485512	0,568127251	0,525758375	0,00000055	0,021
73	PRAD	Prostate	sCpGs	CGGS		21	4	1762	3369	191384	485512	0,523003859	0,394190051	2,2E-51	5,8E-47
74	ESCA	Esophagus	sCpGs	CSGS		21	4	2486	3404	257297	485512	0,730317274	0,529949826	3,3E-126	1,1E-121
75	LIHC	Liver	sCpGs	CGGS		21	4	1669	3336	192465	485512	0,50029976	0,396416566	7,3E-34	1,8E-29
76	BLCA	Bladder	sCpGs	BCCG	**BCGV**	13	4	1996	3425	232947	485512	0,582773723	0,479796586	1,5E-33	4,1E-29
77	BRCA	Breast	sCpGs	SCGS		13	4	2591	3402	238355	485512	0,761610817	0,490935342	9,1E-228	3E-223
78	COAD	Colon	sCpGs	GCGC		13	4	2358	3343	101641	485512	0,705354472	0,20934807	0	0
79	ESCA	Esophagus	uCpGs	KCGR		13	4	2245	3397	271671	485512	0,660877245	0,559555686	2,3E-33	8,8E-29
80	PRAD	Prostate	sCpGs	GCGS		13	4	2122	3369	172467	485512	0,629860493	0,355227059	5,5E-228	1,8E-223
81	COAD	Colon	sCpGs	CCGC		13	4	2238	3343	174394	485512	0,66945857	0,359196065	8,6E-288	2,9E-283
82	LUSC	Lung2	sCpGs	BCGC		13	4	2102	3403	181382	485512	0,617690273	0,373589118	4,2E-181	1,3E-176
83	BLCA	Bladder	uCpGs	AAACRM	**MAASRV**	1	6	343	3401	31127	485512	0,10085269	0,064111701	3,8E-16	1,5E-11
84	THCA	Thyroid	uCpGs	CAASAG		1	6	177	3401	16809	485512	0,052043517	0,034621183	0,00000014	0,0055
85	LIHC	Liver	uCpGs	AGCYCG	**AGCYCS**	20	6	252	3332	25182	485512	0,075630252	0,051866895	4,2E-09	0,00016
86	EPIC	Blood3	sCpGs	AGCTCS		20	6	112	2065	16526	485512	0,054237288	0,034038294	0,0000019	0,045
87	EPIC	Blood3	uCpGs	CTGTGS	**CTGTGS**	11	6	211	2065	34460	485512	0,102179177	0,070976618	0,00000012	0,0041
88	BLCA	Bladder	uCpGs	ACGMAA	**ACGHMR**	3	6	89	3401	6952	485512	0,026168774	0,014318905	0,00000013	0,0045
89	PRAD	Prostate	sCpGs	ACGTCR		3	6	112	3369	7791	485512	0,033244286	0,016046977	2,9E-12	0,00000006
90	BRCA	Breast	sCpGs	CGTCR	**CGBSV**	12	5	176	3402	13935	485512	0,051734274	0,028701659	3,3E-13	6,9E-09
91	UCEC	Uterus2	sCpGs	CGTCR		12	5	190	3374	14240	485512	0,056312982	0,029329862	1,2E-16	2,5E-12
92	ESCA	Esophagus	uCpGs	CGSSG		12	5	1528	3397	146635	485512	0,449808655	0,302021371	6,2E-73	2,5E-68
93	BLCA	Bladder	sCpGs	CGCSS		12	5	2645	3425	154995	485512	0,772262774	0,319240307	0	0
94	BRCA	Breast	sCpGs	CGCSR		12	5	2426	3402	132630	485512	0,713109935	0,273175534	0	0
95	PRAD	Prostate	sCpGs	CGCSR		12	5	2435	3369	132630	485512	0,7227664	0,273175534	0	0
96	EPIC	Blood3	uCpGs	CCTGS	**CCTGS**	10	5	793	2065	157016	485512	0,38401937	0,323402923	3,9E-09	0,00014
97	UCEC	Uterus2	sCpGs	BCGCS	**BCGCS**	35	5	2841	3374	190868	485512	0,842027267	0,393127255	0	0
98	THCA	Thyroid	sCpGs	SCGCS		35	5	2684	3399	164180	485512	0,789644013	0,33815848	0	0
99	LUSC	Lung2	sCpGs	SCGCS		35	5	2869	3403	164180	485512	0,843079636	0,33815848	0	0
100	LIHC	Liver	sCpGs	SCGCS		35	5	2849	3336	164180	485512	0,854016787	0,33815848	0	0
101	HNSC	Head and N	sCpGs	SCGCS		35	5	2809	3361	164180	485512	0,835763166	0,33815848	0	0
102	KIRC	Kidney2	sCpGs	SCGCS		35	5	2771	3297	164180	485512	0,840461025	0,33815848	0	0
103	UCEC	Uterus2	uCpGs	TTWNW	**TTWNW**	24	5	1993	3374	205049	485512	0,590693539	0,422335596	1,9E-85	8,3E-81
104	HNSC	Head and N	uCpGs	ATTCR	**ATTCR**	15	5	223	3360	22432	485512	0,066369048	0,046202772	0,0000001	0,0031
105	KIRC	Kidney2	uCpGs	AKRAA	**AKRAA**	17	5	1049	3297	129061	485512	0,318168032	0,265824532	1,6E-11	0,00000064
106	HNSC	Head and N	uCpGs	AAWGA	**AAWBR**	16	5	315	3360	34311	485512	0,09375	0,070669726	0,00000037	0,011
107	LUSC	Lung2	uCpGs	AAAYR		16	5	932	3385	106570	485512	0,275332349	0,219500239	1,6E-14	6,4E-10
108	THCA	Thyroid	uCpGs	AAAYA		16	5	590	3401	58773	485512	0,173478389	0,121053651	5,4E-19	2,1E-14

Table S3. Cancer tissues considered in this study.

**ABBR.**	**Cancer type**
ACC	Adrenocortical carcinoma
BLCA	Bladder Urothelial Carcinoma
BRCA	Breast invasive carcinoma
CESC	Cervical squamous cell carcinoma and endocervical adenocarcinoma
CHOL	Cholangiocarcinoma
COAD	Colon adenocarcinoma
DLBC	Lymphoid Neoplasm Diffuse Large B-cell Lymphoma
ESCA	Esophageal carcinoma
GBM	Glioblastoma multiforme
HNSC	Head and Neck squamous cell carcinoma
KICH	Kidney Chromophobe
KIRC	Kidney renal clear cell carcinoma
KIRP	Kidney renal papillary cell carcinoma
LAML	Acute Myeloid Leukemia
LGG	Brain Lower Grade Glioma
LIHC	Liver hepatocellular carcinoma
LUAD	Lung adenocarcinoma
LUSC	Lung squamous cell carcinoma
MESO	Mesothelioma
OV	Ovarian serous cystadenocarcinoma
PAAD	Pancreatic adenocarcinoma
PCPG	Pheochromocytoma and Paraganglioma
PRAD	Prostate adenocarcinoma
SARC	Sarcoma
SKCM	Skin Cutaneous Melanoma
STAD	Stomach adenocarcinoma
TGCT	Testicular Germ Cell Tumors
THCA	Thyroid carcinoma
THYM	Thymoma
UCEC	Uterine Corpus Endometrial Carcinoma
UCS	Uterine Carcinosarcoma
UVM	Uveal Melanoma

Table S4. DNA motifs associated with Hyper-iDMC and Hypo-iDMC in cancer tissues. Columns legend: **Tissue ID** - TCGA tissue identification; **Tissue** - tissue name; **Motif** - the identified sequence motif; **Consensus Sequence** - consensus sequences; **Cluster -** motif cluster id; **Length** - length of the identified motif; **Positives** - number of CpGs with the corresponding motif in the input set; **Total positive** - number of total CpGs in the input set; **Negative** - number of CpGs with the corresponding motif in the background set; **Total negative** - Number of total CpGs in the background set; **Positive ratio** - ratio between Positive and Total positive; **Negative ratio** - ratio between Negative and Total negative; **p-value** - motif enrichment P-value; **e-value** - motif enrichment e-value; **Number hyper** - number of Hyper-methylated informative Differentially Methylated CpGs (Hyper-iDMC) in the input set; **Number hypo** - number of Hypo-methylated informative Differentially Methylated CpGs (Hypo-iDMC) in the input set; **Total hyper** - number of total Hyper-methylated informative Differentially Methylated CpGs (Hyper-iDMC) present in the array; **Total hypo** - number of total Hypo-methylated informative Differentially Methylated CpGs (Hypo-iDMC) present in the array; **Motif label** - motif labelled as hyper-methylated if more than 70% of the iDMC carrying the motif were marked as hyper-methylated in the lesion samples as compared to their matched controls, hypo-methylated if more of the 70% of the iDMC carrying the motif were hypo-methylated, undefined otherwise.

Tissue ID	Tissue	Motif	Consensus Sequence	Cluster	Length	Positive	Total positive	Negative	Total negative	Positive ratio	Negative ratio	p-value	e-value	Number hyper	Number hypo	Total hyper	Total hypo	Motif label
PAAD	Pancreatic adenocarcinoma	SCGA	BCGV	27	4	457	1000	176151	485512	0,457	0,362814925	6,6E-10	0,000014	397	133	649	351	Hyper
STAD	Stomach adenocarcinoma	SCGA		27	4	236	1000	84911	485512	0,236	0,174889601	0,00000062	0,011	580	31	901	99	Hyper
UCEC	Uterine Corpus Endometrial Carcinoma	BCGS		27	4	765	1000	307805	485512	0,765	0,633980211	4,4E-19	1,1E-14	747	166	784	216	Hyper
PAAD	Pancreatic adenocarcinoma	SCGS		27	4	708	1000	258736	485512	0,708	0,532913708	9,9E-30	2,5E-25	592	180	649	351	Hyper
STAD	Stomach adenocarcinoma	GCGS		27	4	530	1000	163336	485512	0,53	0,336420109	3,3E-36	7,6E-32	782	11	901	99	Hyper
ESCA	Esophageal carcinoma	CBCGA	CBCGA	12	5	210	1000	68674	485512	0,21	0,141446555	2,7E-09	0,000073	166	67	562	438	Hyper
LUSC	Lung squamous cell carcinoma	CBCGA		12	5	85	1000	23350	485512	0,085	0,048093559	0,00000049	0,014	18	67	223	777	Hypo
SKCM	Skin Cutaneous Melanoma	CBCGA		12	5	449	1000	169003	485512	0,449	0,348092323	3E-11	8,3E-07	121	328	317	683	Hypo
TGCT	Testicular Germ Cell Tumors	CBCGA		12	5	441	1000	162090	485512	0,441	0,333853746	1,3E-12	2,8E-08	0	588	0	1000	Hypo
TGCT	Testicular Germ Cell Tumors	CVACAGC	...CMGB	24	7	39	1000	7599	485512	0,039	0,015651518	0,00000039	0,0076	0	52	0	1000	Hypo
LGG	Brain Lower Grade Glioma	CMGC		24	4	729	1000	311487	485512	0,729	0,641563957	2,5E-09	0,000068	477	252	637	363	Undefined
PCPG	Pheochromocytoma and Paraganglioma	CAGK		24	4	591	1000	251474	485512	0,591	0,517956302	0,0000021	0,024	28	675	34	966	Hypo
STAD	Stomach adenocarcinoma	CBCG	....SBCG	29	4	588	1000	207348	485512	0,588	0,427070804	1,3E-24	2,6E-20	802	54	901	99	Hyper
TGCT	Testicular Germ Cell Tumors	CGACSTCG		29	8	9	1000	312	485512	0,009	0,000642621	0,000000032	0,00062	0	11	0	1000	Hypo
COAD	Colon adenocarcinoma	HSCGS	HSSV..	10	5	864	1000	270863	485512	0,864	0,557891463	2,2E-96	5,6E-92	845	19	947	53	Hyper
UCS	Uterine Carcinosarcoma	CGSGS		10	5	485	1000	172291	485512	0,485	0,354864555	2,5E-17	6,7E-13	305	180	538	462	Undefined
PRAD	Prostate adenocarcinoma	CGCGGWC		10	7	33	1000	4889	485512	0,033	0,010069782	7,1E-09	0,00019	28	5	546	454	Hyper
ESCA	Esophageal carcinoma	CGCGSR		10	6	182	1000	41098	485512	0,182	0,084648783	1,7E-22	4,9E-18	174	8	562	438	Hyper
LUAD	Lung adenocarcinoma	CGCGVA		10	6	125	1000	24354	485512	0,125	0,050161479	2,8E-20	8E-16	122	3	498	502	Hyper
PAAD	Pancreatic adenocarcinoma	BCGCSG	.BVGCN.	6	6	341	1000	76082	485512	0,341	0,156704675	1,4E-46	3,9E-42	317	24	649	351	Hyper
BRCA	Breast invasive carcinoma	GCGCGB		6	6	143	1000	37000	485512	0,143	0,076208209	5,3E-13	1,5E-08	141	2	569	431	Hyper
LUAD	Lung adenocarcinoma	GMGCGS		6	6	154	1000	47945	485512	0,154	0,098751421	0,00000003	0,00083	189	18	498	502	Hyper
STAD	Stomach adenocarcinoma	BSSGCG		6	6	620	1000	133078	485512	0,62	0,274098272	6,8E-115	1,8E-110	611	9	901	99	Hyper
CHOL	Cholangiocarcinoma	BCGCR		6	5	522	1000	152090	485512	0,522	0,313256933	1,5E-42	4,3E-38	508	14	885	115	Hyper
CESC	Cervical squamous cell carcinoma and endocervical adenocarcinor	BCGCK		6	5	697	1000	162235	485512	0,697	0,3341524	7,4E-121	2E-116	691	6	966	34	Hyper
UCEC	Uterine Corpus Endometrial Carcinoma	BCGCK		6	5	546	1000	162235	485512	0,546	0,3341524	6,3E-43	1,7E-38	482	64	784	216	Hyper
DLBC	Lymphoid Neoplasm Diffuse Large B-cell Lymphoma	CGAAATB	CGMWAYB.	7	7	26	1000	2901	485512	0,026	0,005975136	1,1E-09	0,000031	4	22	289	711	Hypo
ESCA	Esophageal carcinoma	CGCTAYCG		7	8	9	1000	365	485512	0,009	0,000751784	0,00000012	0,003	9	0	562	438	Hyper
CESC	Cervical squamous cell carcinoma and endocervical adenocarcinor	CGC	CGV	7	3	701	1000	257577	485512	0,701	0,530526537	3,4E-28	7,8E-24	906	21	966	34	Hyper
CHOL	Cholangiocarcinoma	CGC		7	3	634	1000	264998	485512	0,634	0,545811432	0,00000001	0,00026	779	49	885	115	Hyper
BRCA	Breast invasive carcinoma	CGR		7	3	940	1000	430512	485512	0,94	0,886717527	5,9E-09	0,00016	559	391	569	431	Undefined
COAD	Colon adenocarcinoma	CGS		7	3	464	1000	187427	485512	0,464	0,386039892	0,00000032	0,0053	942	45	947	53	Hyper
THYM	Thymoma	TGCW	YGSH	11	4	528	1000	202876	485512	0,528	0,417859909	1,6E-12	4,6E-08	260	268	481	519	Undefined
COAD	Colon adenocarcinoma	CGSM		11	4	626	1000	231504	485512	0,626	0,476824466	2E-21	4,1E-17	905	24	947	53	Hyper
TGCT	Testicular Germ Cell Tumors	CGSW		11	4	809	1000	335461	485512	0,809	0,690942757	2,3E-17	6,1E-13	0	809	0	1000	Hypo
BLCA	Bladder Urothelial Carcinoma	CAYA	MWY.	5	4	399	1000	156271	485512	0,399	0,321868461	0,00000018	0,0038	11	437	77	923	Hypo
OV	Ovarian serous cystadenocarcinoma	CATB		5	4	503	1000	208509	485512	0,503	0,429462094	0,0000018	0,047	7	537	12	988	Hypo
KICH	Kidney Chromophobe	AWYA		5	4	610	1000	213547	485512	0,61	0,439838768	2,8E-27	8,6E-23	9	601	20	980	Hypo
THCA	Thyroid carcinoma	MACW		5	4	690	1000	280576	485512	0,69	0,577897148	2E-13	4,6E-09	97	671	157	843	Hypo
THCA	Thyroid carcinoma	TSAR	WNRN	3	4	721	1000	287700	485512	0,721	0,592570318	2E-17	5,7E-13	99	622	157	843	Hypo
ACC	Adrenocortical carcinoma	TKAW		3	4	529	1000	215142	485512	0,529	0,44312396	0,000000032	0,00091	134	398	356	644	Hypo
LIHC	Liver hepatocellular carcinoma	WGAW		3	4	554	1000	226184	485512	0,554	0,465866961	0,000000015	0,00034	28	693	63	937	Hypo
LIHC	Liver hepatocellular carcinoma	ATRR		3	4	610	1000	216168	485512	0,61	0,445237193	1,1E-25	3,3E-21	12	598	63	937	Hypo
MESO	Mesothelioma	AWAD		3	4	663	1000	251333	485512	0,663	0,517665887	1,1E-20	3,3E-16	90	573	187	813	Hypo
PRAD	Prostate adenocarcinoma	DTAAA	DTWAA	3	5	210	1000	62302	485512	0,21	0,128322266	5E-13	1,4E-08	42	168	546	454	Hypo
UVM	Uveal Melanoma	RTTAA		3	5	97	1000	25684	485512	0,097	0,052900855	0,000000013	0,00037	30	67	278	722	Undefined
KICH	Kidney Chromophobe	TAA	TBA	20	3	291	1000	109908	485512	0,291	0,226375455	0,0000013	0,024	3	529	20	980	Hypo
MESO	Mesothelioma	TBA		20	3	789	1000	347516	485512	0,789	0,715772216	0,00000008	0,0018	141	725	187	813	Hypo
UVM	Uveal Melanoma	CAYGTGA	.CAYGTGA	20	7	32	1000	4734	485512	0,032	0,009750531	0,000000012	0,00033	1	31	278	722	Hypo
UVM	Uveal Melanoma	TCATATGA		20	8	5	1000	84	485512	0,005	0,000173013	0,0000013	0,037	0	5	278	722	Hypo
PCPG	Pheochromocytoma and Paraganglioma	AAACGW	AAACGW	28	6	39	1000	7772	485512	0,039	0,016007843	0,00000067	0,0079	1	56	34	966	Hypo
PCPG	Pheochromocytoma and Paraganglioma	ASA	ABH	17	3	907	1000	403145	485512	0,907	0,830350228	2,8E-12	5,4E-08	28	928	34	966	Hypo
KICH	Kidney Chromophobe	ATW		17	3	503	1000	194567	485512	0,503	0,400746017	4,2E-11	0,000001	7	658	20	980	Hypo
PCPG	Pheochromocytoma and Paraganglioma	ATH		17	3	799	1000	326661	485512	0,799	0,672817562	5,4E-19	1,6E-14	28	771	34	966	Hypo
LIHC	Liver hepatocellular carcinoma	AGACTMA	.DRANTMA	2	7	20	1000	2259	485512	0,02	0,00465282	0,00000011	0,002	3	22	63	937	Hypo
KIRP	Kidney renal papillary cell carcinoma	TGACTMA		2	7	28	1000	3404	485512	0,028	0,007011155	1,6E-09	0,000046	1	27	467	533	Hypo
HNSC	Head and Neck squamous cell carcinoma	TGABTCA		2	7	53	1000	3925	485512	0,053	0,008084249	5,1E-26	1,5E-21	0	53	226	774	Hypo
MESO	Mesothelioma	TGACTCA		2	7	28	1000	2225	485512	0,028	0,004582791	1,1E-13	2,5E-09	0	31	187	813	Hypo
LIHC	Liver hepatocellular carcinoma	GKAACTCA		2	8	10	1000	676	485512	0,01	0,001392345	0,0000022	0,041	0	11	63	937	Hypo
ACC	Adrenocortical carcinoma	RTGACTCA		2	8	17	1000	1373	485512	0,017	0,002827942	9,2E-09	0,00026	0	17	356	644	Hypo
ESCA	Esophageal carcinoma	MTGASTCA		2	8	26	1000	1869	485512	0,026	0,003849544	9,2E-14	2,5E-09	0	26	562	438	Hypo
BLCA	Bladder Urothelial Carcinoma	RTGAVTCA		2	8	62	1000	2630	485512	0,062	0,005416962	1,8E-43	5,1E-39	0	62	77	923	Hypo
PAAD	Pancreatic adenocarcinoma	ATGASTCA		2	8	14	1000	857	485512	0,014	0,001765147	6,5E-09	0,00012	0	14	649	351	Hypo
MESO	Mesothelioma	GGWGGTA	..DSD..	22	7	17	1000	2092	485512	0,017	0,004308853	0,0000029	0,045	2	15	187	813	Hypo
KIRC	Kidney renal clear cell carcinoma	GWGTCA		22	6	84	1000	16254	485512	0,084	0,03347806	4,9E-14	1,4E-09	5	79	145	855	Hypo
KIRC	Kidney renal clear cell carcinoma	KGTYA		22	5	253	1000	83419	485512	0,253	0,171816557	6,9E-11	0,000002	19	260	145	855	Hypo
OV	Ovarian serous cystadenocarcinoma	GGRCA		22	5	287	1000	100036	485512	0,287	0,206042281	7,9E-10	0,000022	1	286	12	988	Hypo
KICH	Kidney Chromophobe	TGTGW	TGTGW	19	5	143	1000	45981	485512	0,143	0,094706207	0,00000063	0,012	3	217	20	980	Hypo
LGG	Brain Lower Grade Glioma	AARGGTGT	ARDGSDVH	19	8	11	1000	683	485512	0,011	0,001406762	0,00000031	0,0072	9	2	637	363	Hyper
UCS	Uterine Carcinosarcoma	AGKGCAGC		19	8	21	1000	2888	485512	0,021	0,00594836	0,0000012	0,03	9	12	538	462	Undefined
LAML	Acute Myeloid Leukemia	AGGGCTSA		19	8	18	1000	1890	485512	0,018	0,003892798	0,00000016	0,0042	8	11	408	592	Undefined
UCS	Uterine Carcinosarcoma	AGGGCGAA		19	8	9	1000	483	485512	0,009	0,000994826	0,0000011	0,028	8	1	538	462	Hyper
BLCA	Bladder Urothelial Carcinoma	MWGA	.DGA	4	4	802	1000	341449	485512	0,802	0,703276129	8,5E-13	2,4E-08	50	773	77	923	Hypo
KIRC	Kidney renal clear cell carcinoma	AKGA		4	4	515	1000	211618	485512	0,515	0,435865643	0,00000031	0,0082	73	474	145	855	Hypo

OV	Ovarian serous cystadenocarcinoma	ATGAYCGC	ATGABS.	4	8	6	1000	111	485512	0,006	0,000228625	0,00000019	0,0044	0	6	12	988	Hypo
LUAD	Lung adenocarcinoma	ATGACWC		4	7	23	1000	2819	485512	0,023	0,005806242	0,000000052	0,0014	4	19	498	502	Hypo
LUSC	Lung squamous cell carcinoma	ATGASTC		4	7	23	1000	2603	485512	0,023	0,00536135	0,000000013	0,00037	1	22	223	777	Hypo
LUSC	Lung squamous cell carcinoma	ATGWTG	RTGDTG.	16	6	46	1000	10170	485512	0,046	0,020946959	0,0000011	0,032	4	43	223	777	Hypo
HNSC	Head and Neck squamous cell carcinoma	GTGRTGA		16	7	34	1000	4615	485512	0,034	0,009505429	4,9E-10	0,000014	1	37	226	774	Hypo
LUSC	Lung squamous cell carcinoma	RTGGTGA		16	7	34	1000	4765	485512	0,034	0,009814382	1,1E-09	0,000031	2	32	223	777	Hypo
SARC	Sarcoma	GRGAA	RRGVD	23	5	264	1000	96039	485512	0,264	0,197809735	0,00000025	0,0068	68	207	284	716	Hypo
LAML	Acute Myeloid Leukemia	AGGVA		23	5	380	1000	140469	485512	0,38	0,289321376	4,6E-10	0,000013	129	251	408	592	Undefined
TGCT	Testicular Germ Cell Tumors	AGGAK		23	5	160	1000	53582	485512	0,16	0,110361845	0,0000013	0,026	0	236	0	1000	Hypo
LIHC	Liver hepatocellular carcinoma	ARMAT	ADVAB…	26	5	223	1000	67064	485512	0,223	0,138130468	2,8E-13	6,8E-09	4	297	63	937	Hypo
SARC	Sarcoma	ATGAB		26	5	202	1000	61871	485512	0,202	0,127434543	2,7E-11	7,8E-07	26	176	284	716	Hypo
SKCM	Skin Cutaneous Melanoma	ATCGMGAT		26	8	7	1000	238	485512	0,007	0,000490204	0,00000096	0,024	1	6	317	683	Hypo
LAML	Acute Myeloid Leukemia	AMACCAC	AVRHSAM	18	7	29	1000	4653	485512	0,029	0,009583697	0,00000029	0,0074	5	24	408	592	Hypo
KICH	Kidney Chromophobe	AGGTSAC		18	7	32	1000	5238	485512	0,032	0,010788611	0,00000011	0,0021	0	33	20	980	Hypo
LUSC	Lung squamous cell carcinoma	ASGACAA		18	7	23	1000	3111	485512	0,023	0,006407669	0,00000028	0,0082	3	20	223	777	Hypo
UCEC	Uterine Corpus Endometrial Carcinoma	AGCTTAMA	ASCTTAMA	30	8	9	1000	527	485512	0,009	0,001085452	0,0000023	0,044	7	2	784	216	Hyper
UCS	Uterine Carcinosarcoma	ASCTTAAA		30	8	10	1000	593	485512	0,01	0,001221391	0,0000007	0,018	5	5	538	462	Undefined
STAD	Stomach adenocarcinoma	ARCCGAAC	DRCSGAAM	30	8	12	1000	479	485512	0,012	0,000986587	7,8E-10	0,000013	16	0	901	99	Hyper
UCEC	Uterine Corpus Endometrial Carcinoma	KACGGAAA		30	8	11	1000	797	485512	0,011	0,001641566	0,0000013	0,026	9	2	784	216	Hyper
STAD	Stomach adenocarcinoma	AACGGCCG	AMVBGCCB	9	8	6	1000	106	485512	0,006	0,000218326	0,00000015	0,0023	8	0	901	99	Hyper
CHOL	Cholangiocarcinoma	ACSTGCCC		9	8	17	1000	2000	485512	0,017	0,004119363	0,0000016	0,035	16	1	885	115	Hyper
HNSC	Head and Neck squamous cell carcinoma	ACASGCCT		9	8	16	1000	1508	485512	0,016	0,003105999	0,00000019	0,0055	0	16	226	774	Hypo
LUAD	Lung adenocarcinoma	CGCAGCGS	MBVMRDG.	25	8	20	1000	2388	485512	0,02	0,004918519	0,00000025	0,0069	24	0	498	502	Hyper
LUSC	Lung squamous cell carcinoma	CCCCGWGG		25	8	23	1000	3281	485512	0,023	0,006757814	0,0000007	0,02	2	21	223	777	Hypo
LGG	Brain Lower Grade Glioma	ATGCAGGG		25	8	11	1000	622	485512	0,011	0,001281122	0,00000012	0,0029	7	4	637	363	Undefined
THCA	Thyroid carcinoma	ASAAGGG		25	7	26	1000	3818	485512	0,026	0,007863863	0,00000023	0,0043	3	28	157	843	Hypo
ACC	Adrenocortical carcinoma	CAAGGNCA	CMAGKNC.	1	8	44	1000	3307	485512	0,044	0,006811366	1,4E-21	4E-17	1	43	356	644	Hypo
THYM	Thymoma	CCAGKTC		1	7	35	1000	6779	485512	0,035	0,01396258	0,0000013	0,033	13	22	481	519	Undefined
ESCA	Esophageal carcinoma	CGTSGC	VVTBGC.	14	6	74	1000	19938	485512	0,074	0,041065926	0,0000014	0,036	55	30	562	438	Undefined
OV	Ovarian serous cystadenocarcinoma	CAMTGGC		14	7	35	1000	5576	485512	0,035	0,011484783	0,000000015	0,0004	0	35	12	988	Hypo
GBM	Glioblastoma multiforme	GCTGGCY		14	7	57	1000	12794	485512	0,057	0,026351563	0,0000001	0,0028	13	44	336	664	Hypo
UCS	Uterine Carcinosarcoma	CGATKGCC		14	8	11	1000	649	485512	0,011	0,001336733	0,00000019	0,0047	6	5	538	462	Undefined
LUAD	Lung adenocarcinoma	CCACDTTA	SCRCDYTM	13	8	12	1000	839	485512	0,012	0,001728073	0,00000031	0,0084	4	8	498	502	Undefined
ESCA	Esophageal carcinoma	GCGCACTM		13	8	10	1000	601	485512	0,01	0,001237868	0,00000079	0,02	13	0	562	438	Hyper
UCS	Uterine Carcinosarcoma	GCGCACTA		13	8	6	1000	165	485512	0,006	0,000339847	0,0000018	0,044	6	0	538	462	Hyper
CESC	Cervical squamous cell carcinoma and endocervical adenocarcinon	CCGAM	…CMGMM...	8	5	162	1000	45345	485512	0,162	0,09339625	5,9E-12	1,1E-07	174	4	966	34	Hyper
PRAD	Prostate adenocarcinoma	CGWCCGAA		8	8	8	1000	267	485512	0,008	0,000549935	0,00000014	0,0038	8	0	546	454	Hyper
LUAD	Lung adenocarcinoma	CCGAACCC		8	8	13	1000	631	485512	0,013	0,001299659	1,5E-09	0,000043	11	2	498	502	Hyper
LUAD	Lung adenocarcinoma	CAGMCCGC		8	8	18	1000	2146	485512	0,018	0,004420076	0,00000097	0,026	14	6	498	502	Undefined
HNSC	Head and Neck squamous cell carcinoma	CATYCA	..MAYHMR	15	6	63	1000	15273	485512	0,063	0,031457513	0,0000003	0,0085	4	61	226	774	Hypo
GBM	Glioblastoma multiforme	CACAAR		15	6	64	1000	16444	485512	0,064	0,0338694	0,0000016	0,046	10	54	336	664	Hypo
LIHC	Liver hepatocellular carcinoma	CAMATTAA		15	8	9	1000	447	485512	0,009	0,000920678	0,00000061	0,011	0	9	63	937	Hypo
KIRP	Kidney renal papillary cell carcinoma	GCTCAR	RCWMVR..	21	6	92	1000	25759	485512	0,092	0,053055331	0,00000036	0,01	36	56	467	533	Undefined
OV	Ovarian serous cystadenocarcinoma	GCTACATA		21	8	6	1000	168	485512	0,006	0,000346026	0,000002	0,045	0	6	12	988	Hypo
KICH	Kidney Chromophobe	RCTAGA		21	6	34	1000	6638	485512	0,034	0,013672165	0,0000022	0,037	1	34	20	980	Hypo
OV	Ovarian serous cystadenocarcinoma	GCAAGGRA		21	8	13	1000	1149	485512	0,013	0,002366574	0,0000013	0,036	1	12	12	988	Hypo

Table S5. Proportion of TFs associate with hyper- or hypo-methylated motifs divided by classification provided by MethylMinus/MethylMinus classification.

	Hypo-methylated motif	Not characterized motif	Hyper-methylated motif
MethylMinus	0.84	0.06	0.15
MethylPlus	0.71	0.02	0.27
